# β-Cyclocitral, a Master Regulator of Multiple Stress-Responsive Genes in *Solanum lycopersicum* L. Plants

**DOI:** 10.3390/plants10112465

**Published:** 2021-11-15

**Authors:** Shreyas Deshpande, Vishwabandhu Purkar, Sirsha Mitra

**Affiliations:** Department of Botany, Savitribai Phule Pune University, Pune 411007, India; shreyasdeshpande222@gmail.com (S.D.); vdp281298@gmail.com (V.P.)

**Keywords:** apocarotenoids, phytohormone, plant defense, reactive oxygen species, singlet oxygen

## Abstract

β-cyclocitral (βCC), a major apocarotenoid of β-carotene, enhances plants’ defense against environmental stresses. However, the knowledge of βCC’s involvement in the complex stress-signaling network is limited. Here we demonstrate how βCC reprograms the transcriptional responses that enable *Solanum lycopersicum* L. (tomato) plants to endure a plethora of environmental stresses. Comparative transcriptome analysis of control and βCC-treated tomato plants was done by generating RNA sequences in the BGISEQ-500 platform. The trimmed sequences were mapped on the tomato reference genome that identifies 211 protein-coding differentially expressed genes. Gene ontology and Kyoto Encyclopedia of Genes and Genomes analysis and their enrichment uncovered that only upregulated genes are attributed to the stress response. Moreover, 80% of the upregulated genes are functionally related to abiotic and biotic stresses. Co-functional analysis of stress-responsive genes revealed a network of 18 genes that code for heat shock proteins, transcription factors (TFs), and calcium-binding proteins. The upregulation of jasmonic acid (JA)-dependent TFs (*MYC2*, *MYB44*, *ERFs*) but not the JA biosynthetic genes is surprising. However, the upregulation of *DREB3*, an abscisic acid (ABA)-independent TF, validates the unaltered expression of ABA biosynthetic genes. We conclude that βCC treatment upregulates multiple stress-responsive genes without eliciting JA and ABA biosynthesis.

## 1. Introduction

*Solanum lycopersicum* L. (tomato) is one of the most important cultivated food crops in the family Solanaceae [1]. Its productivity and quality are often challenged by various environmental stresses [2]. The extent of damage is dependent on the intensity and duration of the stress to which plants are exposed [3]. However, irrespective of the types of stress and their extent, the generation of reactive oxygen species (ROS) is one of the common hypersensitive responses in stress-exposed plants [4]. Although ROS act as a stress signal, overproduction of these causes phytotoxicity [5]. Therefore, plants have evolved several strategies to detoxify and quench ROS. Plants possess elaborate antioxidant machinery comprised of enzymatic (catalases, superoxide dismutase, peroxidases, etc.) and non-enzymatic (ascorbic acid, proline, carotenoids, flavonoids, etc.) ROS scavengers [6] that act on superoxide radical (O^•−^_2_), hydroxyl radical (OH^•^), hydrogen peroxide (H_2_O_2_), and singlet oxygen (^1^O_2_). Among these, ^1^O_2_ is known as the major ROS generated by photooxidative stress and is responsible for the loss of photosystem I and II activity that ultimately induces cell death [6]. However, recent studies have shown that ^1^O_2_ is also produced after drought and herbivory [3,7,8]. Carotenoids play a prime role in ^1^O_2_ scavenging. Oxidative cleavage of β-carotene produces a number of short-chain volatiles commonly known as apocarotenoids [9]. Interestingly, β-cyclocitral (βCC) is the most abundant apocarotenoid of β-carotene [10] that accumulates under high light, drought, and herbivory [7,9,11]. In addition, simulated herbivory and jasmonic acid (JA) application also resulted in increased accumulation of βCC [11,12]. Recent studies showed that exogenous application of βCC primes plants against drought and develops resistance against insect herbivores [3,11]. Together, these findings indicate that, presumably, βCC is capable of eliciting multiple stress signals. It is known that a large-scale reprogramming in plants’ transcriptome tweaks their proteome and metabolome to survive under unfavorable conditions. In *Arabidopsis thaliana* (Arabidopsis), Ramel et al., (2012) showed that the exogenous application of βCC can regulate the expression of ^1^O_2_-responsive genes mainly responsible for protecting against photooxidative stress [10]. However, βCC’s possible role in multiple stress regulation, especially in commercially important crops, is not explored yet.

In recent years, transcriptome-wide studies by high-throughput sequencing are extensively used to get new insights on genes and their regulatory mechanisms in various plant species [13,14]. The high-throughput sequencing utilizes the principle of next-generation sequencing to annotate transcriptomes in a real-time manner. It enables the detection of both known and novel transcripts along with alternatively spliced isoforms, splice sites, and small and non-coding RNAs [15]. However, RNA sequence analyses of the genome-wide responses of genes to βCC in plants are not known yet. The tomato pan-genome contains over 40,000 protein-coding genes; however, from a large number of genes present, only a few are characterized [16]. Therefore, a comparative analysis of its transcriptome by mapping it with the entire genome can reveal the genome-wide responses to βCC treatment [17]. In the current study, we investigated βCC-induced responses by comparative transcriptome analysis of exogenous βCC-treated and control leaves using RNA-sequencing technology and revealed βCC’s role in developing multiple stress resistance in tomato plants.

## 2. Results

### 2.1. Effect of Exogenous βCC Treatment on Transcriptomic Profile of Tomato Plant

**Figure 1:**
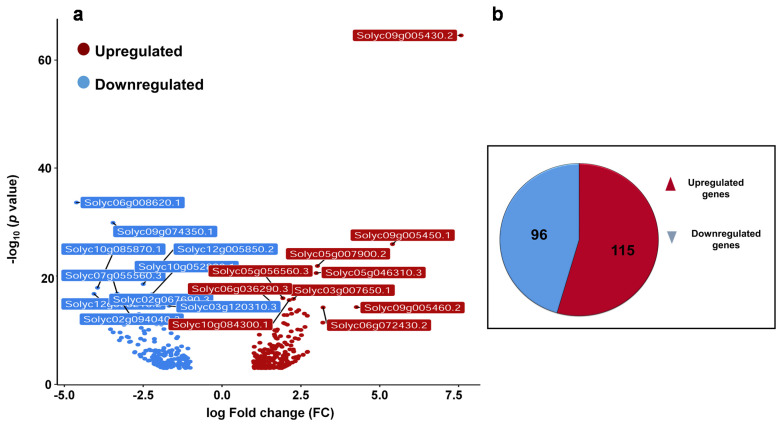
Exogenous application of βCC reprograms transcriptome of tomato plants. Five-week-old tomato plants were treated with βCC or water (control) independently for 4 h, and harvested samples were subjected to RNA sequencing. The obtained transcriptome was tailored with several statistical parameters to get the most differentially expressed genes (DEGs). (**a**) A volcano plot was generated with the 412 DEGs to depict upregulated (red dots) and downregulated (blue dots) genes. Statistically most significant genes are marked with their gene identification (ID) numbers. (**b**) Further filtering of these 412 DEGs, 211 protein-coding DEGs are obtained, out of which 115 DEGs are upregulated (log_2_FC > 1) and 96 are downregulated (log_2_FC < −1).

Previously, it was shown that βCC treatment enhances resistance to high light stress and regulates singlet oxygen-responsive genes. Recently, we have seen that in addition to high light, βCC treatment also develops resistance to drought stress and herbivory in tomato and Arabidopsis plants, respectively [3,10,11]. Therefore, we hypothesized that βCC plays a pivotal role in the regulation of multiple stress responses. To see how it orchestrates the multiple defense responses, we analyzed the transcriptome of βCC-treated tomato plants and compared it with the control plants. The volcano plot (Figure 1a) shows that in total, 412 genes are differentially expressed after βCC treatment (1 ≤ log_2_ fold change ≤ −1 at *p* ≤ 0.05). Among these 412 genes, 211 genes are protein-coding differentially expressed genes (DEGs) where the number of upregulated genes (115) is greater than the downregulated genes (96) (Figure 1b). A list of protein-coding DEGs is provided in Supplementary Data S1. As the remaining 201 DEGs are not coding for any protein, we excluded them from the analysis.

### 2.2. Validation of Differentially Expressed Transcripts by Quantitative Real-Time PCR

**Figure 2:**
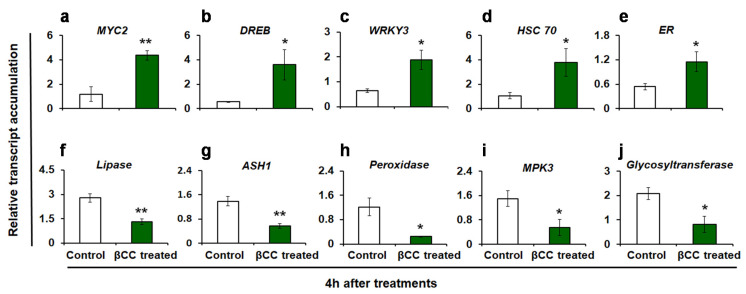
Validation of RNA sequencing results by quantitative real-time (q-RT) PCR. Relative transcript accumulations of ten candidate genes were evaluated by q-RT PCR. Similar to the RNA sequencing data, in βCC-treated plants, the relative transcript accumulations of (**a**) *MYC2*, (**b**) *DREB*3, (**c**) *WRKY3*, (**d**) *HSC70*, and (**e**) *ER* are significantly higher and transcript accumulations of (**f**) *lipase*, (**g**) *ASH1*, (**h**) *peroxidase*, (**i**) *MPK3*, and (**j**) *glycosyltransferase* are significantly lower as compared to control plants. Values are the mean ± SE of four replicate plants from each treatment. Significant differences are determined with Student’s *t*-test. Asterisks indicate significance at * *p* ≤ 0.05, ** *p* ≤ 0.005. *Dehydration responsive element-binding 3*, *DREB3*; *Heat shock cognate 70*, *HSC 70*; *Ethylene receptor*, *ER*; *Acylsugar acylhydrolase* 1, *ASH1*; *Mitogen-activated protein kinase 3*, *MPK3*.

The reproducibility of the DEGs obtained from RNA sequencing was validated by quantitative real-time (q-RT) PCR. The expression of 10 randomly selected transcripts (including both up- and downregulated transcripts) was assessed (Figure 2). Similar to the RNA sequence data, the transcript accumulations of *MYC2* (*p* = 0.002) (Figure 2a), *dehydration-responsive element-binding* 3 (*DREB*3) (*p* = 0.024) (Figure 2b), *WRKY3* (*p* = 0.01) (Figure 2c), *heat shock cognate 70* (*HSC70*) (*p* = 0.027) (Figure 2d), and *ethylene receptor* (*ER*) (*p* = 0.027) (Figure 2e) are increased in βCC-treated plants as compared to control. On the other hand, the transcript accumulations of *lipase* (*p* = 0.001) (Figure 2f), *acylsugar acylhydrolase 1* (*ASH1*) (*p* = 0.001) (Figure 2g), *peroxidase* (*p* = 0.007) (Figure 2h), *mitogen-activated protein kinase 3* (*MPK3*) (*p* = 0.022) (Figure 2i), and *glycosyltransferase* (*p* = 0.011) (Figure 2j) are decreased as compared to control plants. Similar trends in the expression of selected transcripts in q-RT PCR and RNA sequencing endorse the reliability of the RNA sequence data.

### 2.3. βCC-Induced Differentially Expressed Genes (DEGs) Are Mainly Coding the Proteins That Are Regulated by External Stimuli

**Figure 3:**
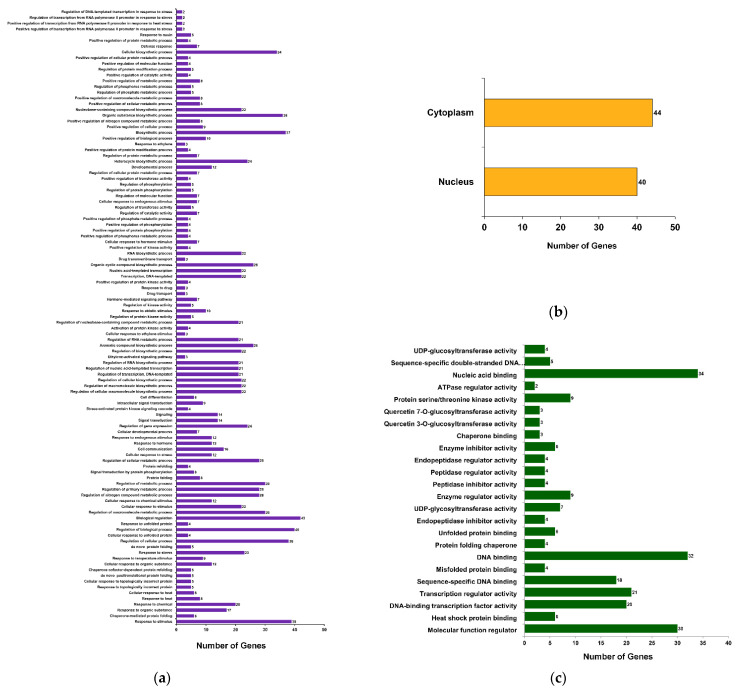
Functional classification of DEGs based on gene ontology (GO). DEGs are annotated with different GO terms and categorized into 133 functional groups. Among these, (**a**) 107 groups are categorized under GO category biological process (GOBP), (**b**) two are under GO category cellular component (GOCC), and (**c**) 24 groups are categorized under GO category molecular function (GOMF).

To understand the biological significance of the βCC-mediated DEGs, gene ontology (GO) analysis was performed. A total of 352 GO terms were obtained; among these, 159, 39, and 154 terms belong to the GO categories biological process (GOBP), cellular component (GOCC), and molecular function (GOMF), respectively (Supplementary Data S2). Annotation frequencies of DEGs revealed that most of the genes were annotated with the GOCC term nucleus (GO:0005634), with 43 genes having this annotation. Similarly, 25 genes were annotated with the GOMF term DNA binding (GO:0003677) and 23 genes were annotated with the GOBP term regulation of transcription, DNA-templated (GO:0006355) (Supplementary Data S2). To comprehend this large number of GO annotations, they are subjected to statistical analysis and significantly annotated terms (at FDR < 0.05) are categorized into different functional groups, where GOBP has 107 (Figure 3a), GOCC has 2 (Figure 3b), and GOMF has 24 functional groups (Figure 3c). Interestingly, the maximum number of genes were annotated with GOBP terms belonging to the group biological regulation (GO:0065007; 42 genes). Correspondingly, functional groups cytoplasm (GO:0005737; 44 genes) and nucleic acid binding (GO:0003676; 34 genes) show more genes as compared to the other functional groups (Supplementary Data S2). This suggests that βCC treatment significantly regulates a large number of genes related to the regulation of biological processes.

### 2.4. Upregulated Genes Are Significantly Enriched with Stress-Responsive Functions

Though GO annotations indicate the involvement of βCC-induced DEGs in stress response, whether it is governed by up- or downregulated genes was not clear. Therefore, GO enrichment analyses of up- and downregulated genes were done independently. Upregulated genes were distributed into different functional groups where GOBP, GOCC, and GOMF had 121, 2, and 28 significant functional groups (enrichment FDR < 0.05), respectively. Similarly, in down-regulated genes, GOBP, GOCC, and GOMF had 3, 2, and 16 significant (enrichment FDR < 0.05) functional groups, respectively (Supplementary Data S3). These significant functional groups from up- and downregulated genes were ranked according to their FDR values (Supplementary Data S3).

**Figure 4:**
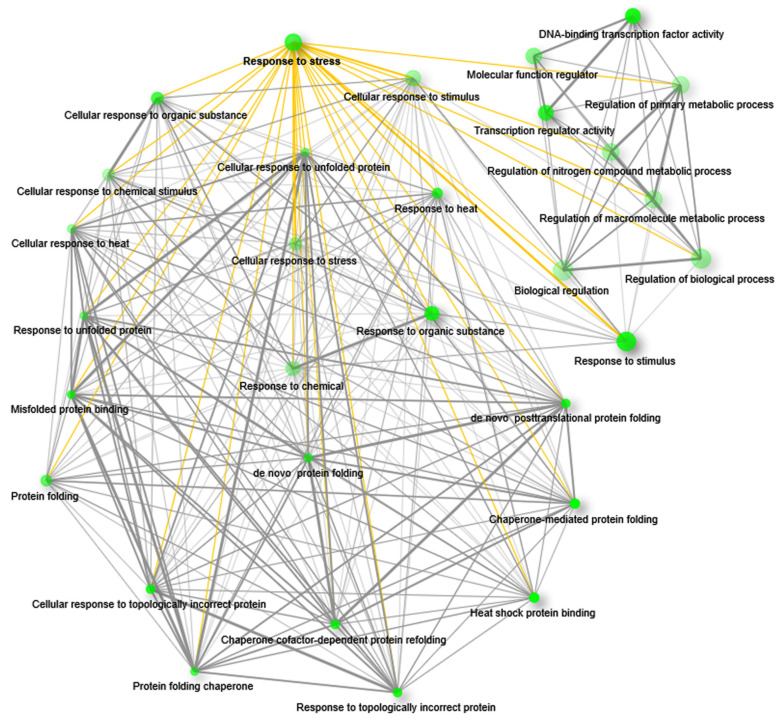
GO enrichment and interrelationship of the functional groups. GO enrichment of upregulated genes showed functional interrelationship among different functional groups. The network represents the top 30 significant functional groups at an enrichment FDR < 0.05. The network showed intricate interrelations among different functional groups including the functional group response to stress. Green nodes represent the functional groups. The size of the node is proportional to the number of genes and darkness of the nodes is proportional to the enrichment value at FDR < 0.05. Grey lines (edges) that are joining the nodes are showing functional interrelations between the nodes. The yellow edges are highlighting the interrelations of the group response to stress with other functional groups. For better visualization the positions of the nodes were rearranged.

The GO enrichment network was created with upregulated genes that were annotated with all possible terms; this resulted in different functional groups, where ‘response to stress’ (RS) is one of the major ones (Figure 4; Supplementary Data S3). The group RS is present among the top-ranked functional groups according to its FDR value (Supplementary Data S3) and is connected to the 26 different functional groups in the network (Figure 4; Supplementary Data S3). Interestingly, connected functional groups are also related to defense responses. Among them, heat shock protein binding (GO:0031072; edge width = 0.34626) and chaperone-mediated protein refolding (GO:0061077; edge width = 0.2) are the most enriched functional groups from the GOBP and GOMF category, respectively. In addition, RS is also connected with other groups such as response to stimulus, response to organic substance, cellular response to stress, protein folding, etc. (Supplementary Data S3). On the other hand, the network of functional groups associated with downregulated genes did not show the functional group RS (Supplementary Figure S1). Here, the functional group drug transport (GO:0042908) was the most enriched functional group (FDR = 0.008352535) from the GOBP category, and integral component of membrane (GO:0016021) and intrinsic component of membrane (GO:0031224; FDR = 0.0229) were the most enriched from the GOCC category. From the GOMF category, the UDP-glycosyltransferase activity group (GO:0008194; FDR = 0.000601355) was the most enriched functional group (Supplementary Data S3).

Enrichment analysis revealed that only the upregulated genes are annotated with GO terms belonging to the group RS. This group is functionally associated with other top-ranked functional groups that are related to stress responses. Moreover, the functional groups associated with the highest number of genes are also connected with RS in the network. Therefore, we infer that the upregulated genes are mainly governing the defense responses to environmental stresses.

### 2.5. Exogenous βCC Treatment Specifically Upregulates Genes That Are Involved in Acclimation to the Plethora of Environmental Stresses

**Figure 5:**
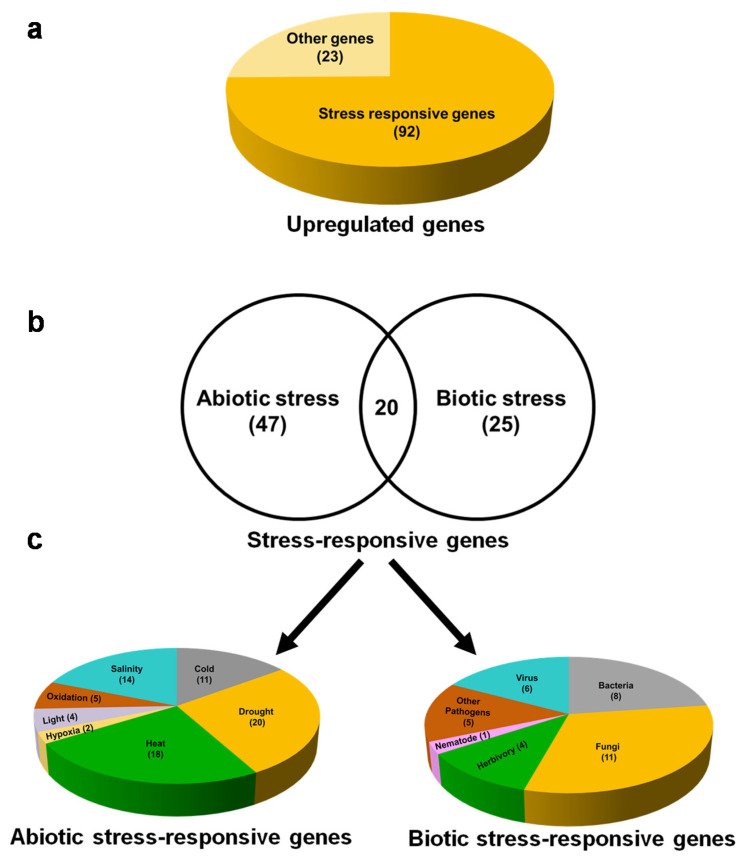
Exogenous βCC treatment mainly upregulates stress-responsive genes. Among the 115 upregulated genes, (**a**) 92 genes are involved in stress response, and only 23 genes are performing other functions. Among these 92 upregulated stress-responsive genes, (**b**) 47 and 25 genes are specific to abiotic and biotic stress, respectively. There are 20 genes that are commonly involved in both abiotic and biotic stress responses. (**c**) Abiotic stress-responsive genes are related to drought, heat, salinity, hypoxia, oxidative stress, light, and cold (left panel); on the other hand, biotic stress-responsive genes are related to herbivory, fungi, bacteria, nematode, viruses, and other pathogens (right panel). A few genes that are involved in multiple stresses are counted under each of those respective stresses; therefore, the total numbers of abiotic and biotic stress-responsive genes are more than 47 and 25, respectively.

To reveal the role of 115 upregulated genes, functional predictions of the DEGs were carried. Interestingly, among 115 upregulated genes, 92 (80%) are stress-responsive (Figure 5a) and 23 genes are related to other functions. We found that, among the stress-responsive genes, 47 and 25 genes were specifically responsible for abiotic and biotic stresses, respectively. However, 20 genes have proven a role for both abiotic and biotic stresses (Figure 5b; Supplementary Data S4). A total of 92 DEGs that are responsive to abiotic and biotic stresses are involved in more than one stress; therefore, the same DEG is counted for each stress it is involved in. Abiotic stress-responsive DEGs include drought (20), heat (18), salinity (14), cold (11), oxidative (5), light (4), and hypoxia (2) (Figure 5c left panel). Similarly, DEGs responsive to biotic stresses include the attack of fungi (11), bacteria (8), herbivores (4), viruses (6), other pathogens (5), and nematode (1) (Figure 5c right panel). Among abiotic stress-responsive genes, those regulating responses to drought (20 DEGs) and heat (18 DEGs) are high in number. On the other hand, among biotic stress-responsive genes, those regulating responses to fungal (11 DEGs) and bacterial (eight DEGs) attacks are high in number (Supplementary Data S4).

Furthermore, documentation of the molecular classes of stress-responsive genes revealed that most of the DEGs responsive to heat stress are chaperones (12 DEGs). In addition, different transcription factors related to drought (seven DEGs) and fungal (four DEGs) and bacterial attack (three DEGs) are also evident (Supplementary Data S4). All these findings are consistent with the results of GO enrichment analysis. Thus, functional predictions of DEGs aided with their molecular classes indicate that βCC specifically induces the stress-responsive genes to manage the plethora of environmental stresses.

### 2.6. Upregulated DEGs Are Specifically Involved in Pathways for Defense Response

**Figure 6:**
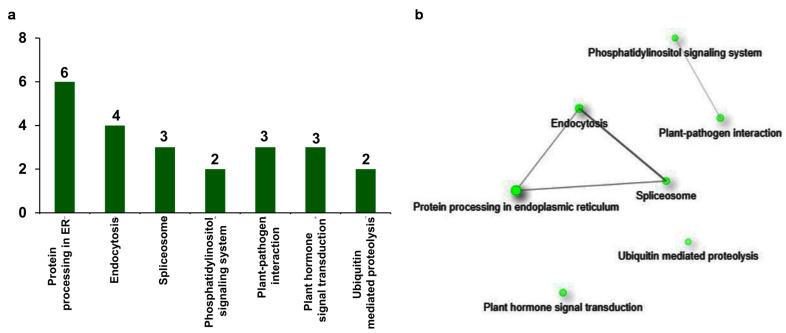
Distribution of stress-responsive genes across KEGG pathways. Among upregulated genes, stress-responsive genes were subjected to KEGG enrichment and network analysis. (**a**) KEGG enrichment analysis of 92 DEGs resulted in 23 annotated DEGs with at least one out of seven KEGG terms. (**b**) Network analysis of stress-responsive DEGs showed that the DEGs attributed to different defense-related pathways. Green nodes represent the functional groups. The size of the node is proportional to the number of genes, and darkness of the node is proportional to the enrichment value at FDR < 0.05. Grey lines (edges) that are joining the nodes are showing functional interrelations between the nodes.

In the functional prediction and GO enrichment analysis of upregulated genes, it is evident that they encode proteins that are responsive to multiple biotic and abiotic stresses. The involvement of these proteins in specific molecular and biochemical pathways is ultimately expressed as stress-responsive phenotype. Therefore, to understand the role of upregulated genes in the regulation of different biological pathways related to stress response, they are also annotated with Kyoto Encyclopedia of Genes and Genomes (KEGG) terms. In the analysis, seven different pathways were detected for 23 genes (Figure 6a). Interestingly, the KEGG term protein processing in endoplasmic reticulum (ER) (sly04141) is the most frequently annotated to six genes, followed by the term endocytosis (sly04144) to four genes. Other KEGG terms spliceosome (sly03040), plant pathogen interaction (sly04626), and plant hormone signal transduction (sly04075) are annotated to three genes, and phosphatidylinositol signaling system (sly04070) is annotated to two genes. The last term found in the analysis is ubiquitin-mediated proteolysis (sly04120), which is annotated to two genes (Figure 6a).

To interpret the correlation between the KEGG terms, KEGG enrichment and network analysis of upregulated stress-responsive genes was conducted. The obtained network represented the KEGG terms as nodes which are connected through the edges (Supplementary Data S5). Among the obtained KEGG terms, the term protein processing in endoplasmic reticulum (ER) (sly04141; FDR = 8.07 × 10^−6^) is the most significant one. This is connected with the terms endocytosis (sly04144; edge width = 0.918367347) and spliceosome (sly03040; edge width = 1.25) (Figure 6b). In addition, the terms endocytosis and spliceosome are also connected to each other (edge width = 2.8125). The terms phosphatidylinositol signaling system (sly04070) and plant–pathogen interaction (sly04626) are forming a separate group and connected to each other in the network (edge width = 0.3125). However, the term plant hormone signal transduction (sly04075) and ubiquitin mediated proteolysis (sly04120) do not show any connection with any of the terms (Figure 6b). In summary, KEGG enrichment analysis of upregulated genes uncovered the stress-responsive pathways that are elicited after βCC treatment.

### 2.7. βCC Treatment Co-Expressed the Transcription Factors and Other Protein-Encoding Genes That Provide Acclimation to Multiple Stresses

**Figure 7:**
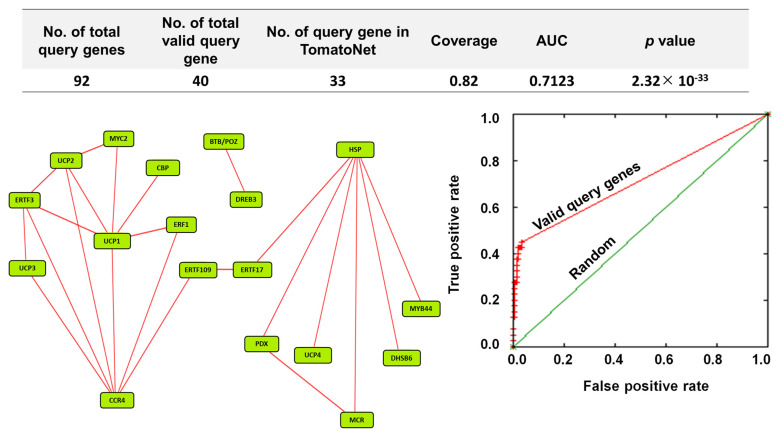
A co-functional network analysis of stress-responsive DEGs validates βCC’s role in the regulation of multiple stress-responsive genes. Top and left panel: Among 92 upregulated stress-responsive DEGs, 40 queries were valid. From these, 33 valid queries were present in TomatoNet. Among them, 18 stress-responsive genes are co-functional. Right panel: Co-functional network is validated by receiver operating characteristics (ROC) curve where the area under curve (AUC) value is 0.7123 at *p* = 2.3 × 10^−33^. For better visualization, the positions of the nodes were rearranged. CCR4, probable CCR4-associated factor 1 homolog 9; HSP, heat shock 70 kDa protein 8; UCP1, uncharacterized protein LOC101261732; UCP2, uncharacterized protein LOC104645686; ERTF3, ethylene-responsive transcription factor 3; UCP3, uncharacterized protein LOC101254906; PDX, pyridoxal 5′-phosphate synthase-like subunit PDX1.2; MCR, BAG family molecular chaperone regulator 5, mitochondrial-like; MYC2, transcription factor MYC2; ERTF17, ethylene-responsive transcription factor ERF017; DHSB, DNAJ homolog subfamily B member 6-like; DREB3, dehydration-responsive element binding protein 3; BTB/POZ, BTB/POZ and MATH domain-containing protein 2-like; UCP4, uncharacterized protein LOC101245195; ERF1, ethylene-responsive transcription factor1; MYB44, transcription factor MYB44; ERTF109, ethylene-responsive transcription factor ERF109-like; CBP, probable calcium-binding protein CML30.

To understand the regulatory mechanism of upregulated genes in orchestrating defense response, 92 stress-responsive upregulated genes were subjected to co-functional analysis on TomatoNet [18]. It was found that out of 92 stress-responsive genes, 40 queries were valid. Out of these valid queries, 33 queries were detected in the predefined network on TomatoNet [18] (Figure 7; upper panel). A total of 18 DEGs were co-functional with the coverage of 0.82 (Figure 7; upper and left panel). The corresponding receiver operating characteristic (ROC) curve showed that the network has an area under curve (AUC) value of 0.7123 at *p* = 2.32 × 10^−33^ (Figure 7; right panel).

Functional annotations of the 18 genes revealed that all are related to defense response (Supplementary Data S2 and S6). The network of these genes exhibits a pivotal node of heat shock 70 kDa protein 8 (Solyc09g075950), which is connected to six other query genes, namely pyridoxal 5′-phosphate synthase-like subunit PDX1.2 (PDX), BAG family molecular chaperone regulator 5, mitochondrial-like (MCR), DNAJ homolog subfamily B member 6-like (DHSB), MYB44, uncharacterized protein LOC101245195 (UCP4), and ethylene-responsive transcription factor ERF017 (ERTF17); similarly, another cluster has probable CCR4-associated factor 1 homolog 9 (CCR4) (Solyc06g074030.1) as a pivotal node which is connected with eight other query genes, namely ethylene-responsive transcription factor 3 (ERTF3), ethylene-responsive transcription factor 1 (ERF1), ethylene-responsive transcription factor 109-like (ERTF109), uncharacterized protein LOC101254906 (UCP3), uncharacterized protein LOC101261732 (UCP1), uncharacterized protein LOC104645686 (UCP2), transcription factor MYC2 (MYC2), and probable calcium-binding protein CML30 (CBP). These two clusters are connected via ERTF109 (Solyc10g050970.1) and ethylene-responsive transcription factor ERF017 (ERTF17) (Solyc12g009240.1). In the network, two nodes, namely DREB3 (Solyc04g072900.1) and BTB/POZ and MATH domain-containing protein 2-like (Solyc09g072980.2), are connected but separated from the rest of the network (Figure 7). The genes involved in this network are representating key transcription factors (TFs), namely DREB3 (Solyc04g072900.1), MYC2 (Solyc08g076930.1), MYB44 (Solyc04g078420.1), ERF3 (Solyc10g006130.1), ERF017 (Solyc12g009240.1), ERF1 (Solyc03g093610.1), and ERF 109-like (Solyc10g050970.1), that are involved in tuning abiotic and biotic stress responses in different plants. Therefore, the co-expression of these key transcription factors confirms βCC’s role in the regulation of multiple stress responses.

## 3. Discussion

To survive under a suboptimal environmental condition, plants adapt their defense strategies depending upon the environmental cues. Therefore, stress sensing and signaling are the prime arsenals of plants during stress. In recent years, apocarotenoids are gaining importance as a stress signal [19,20]. Previous studies showed that βCC is a major apocarotenoid of β-carotene. It can induce ^1^O_2_-responsive genes, which are essential for photooxidative stress [10]. Recently, we found that βCC can also prime plants against drought and develop resistance against herbivory [3,11]. These observations stemmed the hypothesis that βCC induces multiple stress tolerance in plants via reprogramming their transcriptome. Therefore, we compare the transcriptome of βCC-treated plants with control plants to reveal βCC-induced transcriptomic changes in tomato plants. Our results uncover that βCC treatment triggers multiple stress-responsive genes that are essential to counteract both abiotic and biotic stresses. The abiotic stresses include drought, heat, cold, excess salt, heavy metals, and nutrient deficiency, and the biotic stresses include herbivore infestation and pathogen infection. Different stresses are sharing common signal transduction pathways to relay the information, because presumably the same protective measures are required to counteract them [21]. For example, the generation of ROS is a common phenomenon after all the stresses; therefore, the production of antioxidants and ROS-scavenging enzymes are a prerequisite for the protection against oxidative damage triggered by different abiotic and biotic stresses. Similarly, βCC is produced by oxidative cleavage of β-carotene after exposure to photooxidative stress, drought, and herbivores as a result of a common ^1^O_2_-scavenging mechanism; therefore, production of βCC might also be a common signal for multiple stresses.

In the present study, we found that the number of upregulated genes is greater than the downregulated genes (Figure 1), and 80% of upregulated genes are related to stress response (Figure 5). In addition, only upregulated genes are attributed to the GO term stress response (Figure 3). Interestingly, GO and KEGG enrichment analysis revealed that βCC-induced upregulated genes are an integral part of the pathways that attribute to the enhanced stress tolerance. The pathways include protein processing in endoplasmic reticulum (ER), plant hormone signal transduction, endocytosis, spliceosome, phosphatidylinositol signaling system, plant–pathogen interaction, and ubiquitin mediated proteolysis. Based on the microarray analysis, researchers categorized the stress-induced genes into two groups according to the functions of their products [22,23]. The first group consists of functional proteins essential for maintaining the homeostasis in the cell, and the second group consists of regulatory proteins involved in the regulation of signal transduction and gene expression. Similarly, the network of the KEGG-enriched pathways of upregulated genes is also grouped into two categories (Figure 6b) depending on the functions of the DEGs involved in them. The first category includes protein processing in ER, endocytosis, and spliceosome, which are represented by the functional proteins, namely heat shock proteins and heat shock cognates. The second category includes phosphatidylinositol signaling, plant–pathogen interaction, hormone signal transduction, and ubiquitin-mediated proteolysis, which consist of the regulatory proteins, namely calcium-binding proteins, transcription factors, and kinases. These results confirm βCC’s role in reprogramming of the tomato transcriptome.

Researchers have shown that the misfolding of protein or the accumulation of unfolded proteins are likely in plants after being exposed to both biotic and abiotic stress [24,25]. This is sensed by the specific sensor proteins in the ER membrane, and genes that encode chaperones and other proteins are expressed to enhance the capacity of protein folding [26,27]. In our analysis, among the KEGG terms found, the term protein processing in ER was the most frequently annotated term. Moreover, βCC application upregulates the transcript accumulations of heat shock proteins (*HSP70-kD*, *HSP 40-kD*), *heat shock cognate 70* (*HSC70*) (a protein highly homologous to *HSP70* chaperones), and *DNAJ domain-containing protein chaperone*. These chaperones are multifunctional and, in addition to protecting the plants from environmental stresses, also regulate the distribution and activities of proteins involved in developmental and metabolic processes [28,29]. The families of HSPs are collectively known as molecular chaperones and are classified into four major groups based on their approximate molecular weights [30,31]. Each group is localized to one of the compartments, namely cytosol, endoplasmic reticulum (ER), plastids, and mitochondria [32], and performs different functions. For instance, the cytosolic HSP70 in Arabidopsis is involved in enhanced thermotolerance [33], whereas ER HSP70 in *Nicotiana tabacum* enhanced tolerance to drought but not to heat [34]. On the other hand, mitochondrial HSP70 facilitates membrane translocation of preproteins [35], and plastidial HSP70 is believed to be involved in the import of protein into chloroplasts [36], plant development, and in thermotolerance of germinating seeds [37]. However, HSP70/90 is also an important molecular chaperone that plays a critical role in biotic stress responses. The HSC70 is required to induce hypersensitive response (HR) against pathogens, namely *Phytophthora infestans* INF1, *Pseudomonas cichorii* [38], and *Xanthomonas campestris* I [39]. Similarly, HSP90 can activate cytosolic R proteins that mediate defense against many viral pathogens and immunity against fungi [40].

Under environmental stresses, plants recruit an efficient signaling network starting from sensing the external stimulus to the final response. Cytosolic calcium (Ca^2+^) flux, the activity of protein phosphatases and kinases, and several transcription factors (TFs) play a prime role in the process of signal transduction and gene expression regulation. A stress-induced rapid increase in Ca^2+^ works as a second messenger in cellular stress signal transductions [41,42]. Calmodulin-like proteins (CMLs) are the largest class of Ca^2+^ sensors that act as sensor relays to regulate downstream functions in plants [43]. *CML* genes play a vital role in plant–microbe interaction [44]. An increase in *CML-30* in βCC-treated plants indicates that βCC-treated plants anticipate a Ca^2+^ flux and prepare the plants to relay the perceived signal. Ca^2+^ functions parallelly with other important secondary messengers such as ROS. Mitogen-activated protein kinases (MAPKs) respond to ROS bursts caused by various abiotic and biotic factors [45,46]. Generally, a MAPK cascade has three sequentially acting serine/threonine kinases, namely the MAP kinase kinase kinase (MAPKKK), the MAP kinase kinase (MAPKK), and the MAP kinase (MAPK). Activation of a MAPKKK is considered the first step of this cascade. βCC treatment can increase the transcript accumulation of different MAPKKK 1-like proteins in tomato; however, βCC treatment neither causes an increase in ROS accumulation [3] nor is attributed to the KEGG term MAPK pathway. Therefore, presumably, the MAPK pathway does not play a prime role in βCC-mediated defense response.

Plant hormones play a crucial role in plants’ growth, development, and defense responses. Plants produce a bouquet of hormones, namely auxins (AUX), gibberellins (GA), cytokinins (CK), abscisic acid (ABA), ethylene (ET), salicylic acid (SA), jasmonates (JA), brassinosteroids (BR), and strigolactones. Among these, ABA, SA, JA, and ET are the major ones that mediate plant defense response against abiotic stresses and biotic stresses [47]. Surprisingly, transcripts of any of their biosynthetic genes are not regulated after 4 h of βCC treatment. Previous studies showed that βCC treatment did not increase the accumulation of ABA [3,7]; moreover, βCC-induced upregulation of DREB3 transcription factor indicates that βCC treatment regulates the stress responses in an ABA-independent manner. However, upregulation of the JA-, SA-, and ET-dependent transcription factors, namely *MYC*, *WRKY*, *MYB*, *bHLH*, and *ERF-1*, is unexpected.

Accumulated JA is conjugated with free isoleucine (Ile) to form jasmonoyl-L-isoleucine (JA-Ile) in the presence of JASMONATE RESISTANT 1(JAR1) [48,49]. JA-Ile is the bioactive form of JA that favors the interaction between the two co-receptors of jasmonate, JASMONATE ZINC FINGER INFLORESCENCE MERISTEM-DOMAIN (JAZ) and CORONATINE INSENSITIVE-1 (COI-1). COI-1 is an F-box protein that occurs in assembly with proteins SKP1 and Cullin to form SCF-type E3 ubiquitin ligase (SCF^COI1^). The binding of JAZ with COI1 ubiquitylates it in such a fashion that it is targeted for 26S proteasomal degradation. The cleavage of JAZ results in the activation of TFs such as MYCs to expresses JA-dependent defense genes [50]. Similarly, the removal of JAZ also enhances the transcriptional activity of the EIN3/EIL1 transcription factor that induces the expression of ethylene-responsive transcription factor 1 (*ERF1)* [51]. Ethylene-responsive transcription factors (ERFs) are the major downstream regulatory factors in the ET signaling pathway. ERF1 stimulates genes such as *plant defensin 1.2* (*PDF1.2*) and *basic endochitinase B* that play a defensive role against necrotrophic and hemibiotrophic fungal pathogens [51,52]. Therefore, an upregulation of the expression of *ethylene-responsive transcription factor 1* (*ERF1*) and *EIN3*-like 4 protein indicates that βCC treatment induces the ET signaling pathway. However, the absence of JA-mediated degradation of JAZ is incongruent with the increased expression of *MYC2* and *ERF1* that mediates JA- and ET-mediated defense response, respectively [53,54]. Previously, it was shown that α-ionone, another apocarotenoid of β-carotene, can induce the JA-mediated stress response without accumulating JA [55]. This suggests that, like α-ionone, βCC is also capable of inducing the jasmonate-dependent TFs, bypassing the jasmonate signaling; however, the mechanism is not clear. The trend of gene expression without removing the transcriptional repressor is also evident in AUX signaling. βCC treatment increases the accumulation of AUX responsive proteins, namely *small auxin upregulated RNA* (*SAUR 71*) and *AUX-induced proteins 15-like* (homologous to the *SAUR7* gene from *A. thaliana*) [56], without altering the expression of AUX/IAA proteins, a repressor of early auxin response genes’ expression [57]. It is known that in plants, the ubiquitin system is an integral part of hormonal signal transduction. This process is coordinated via three types of enzymes, namely ubiquitin-activating enzyme (E1), ubiquitin-conjugating enzyme (E2), and ubiquitin ligase enzyme (E3) [58]. Mainly, E3 ligases determine the specificity of this system. E3 ligases bind and ubiquitylate specific sets of proteins essential for proteasome activity [59]. Interestingly, KEGG enrichment analysis showed that the KEGG term ubiquitin-mediated proteolysis is represented by ubiquitin-conjugating enzyme E2-2 and an F-box protein S-Phase Kinase-Associated Protein2B (SKP2B). Interestingly, SKP2B functions as E3 ligase [60]. This suggests that βCC might trigger a different ubiquitin system that works independently of JA, ET, and AUX.

SA is another important phytohormone mainly involved in the regulation of plant defense against pathogens [61]. It was shown that βCC treatment enhances SA accumulation in Arabidopsis [62]; however, we did not find upregulation of the genes involved in SA biosynthesis (isochorismate synthase) nor of the downstream genes, which are regulated by SA (nonexpressor of pathogenesis-related genes 1 (NPR1); TGACG-Binding (TGA) transcription factors). Lv et al., (2015) showed that SA accumulation occurs after 6 h of the βCC treatment [62]; therefore, SA biosynthesis and SA-induced genes may be regulated later, and hence their regulation is not captured in the current study.

Abiotic and biotic stresses not only induce defenses but also cause the suppression of growth [63]. This happens because plants redirect the resources from growth to defense when threatened by different stresses [64]. Therefore, plants recruit different strategies to maintain a balance between growth and defense. Recently, we found in Arabidopsis that exogenous application of βCC while increasing defense against herbivores, suppressing flux through the methylerythritol 4 phosphate (MEP) pathway, a pathway involved in growth and carbon assimilation [11]. Our results indicate that βCC treatment can induce the genes required for multiple defense responses by detouring the phytohormone signaling. This could be another strategy to maintain a balance between growth and defense.

The co-functional network validated the abiotic and biotic stress-responsive traits that are controlled by βCC. Eighteen query genes form three clusters, where one cluster consists of genes responsible for biotic stresses and the second one consists of genes responsible for abiotic stresses. Interestingly, these two clusters are connected by *ethylene-responsive transcription factor 1*, which encodes ERFs. This highlights that the ET signaling is presumably coordinated between abiotic and biotic stress response. The third cluster has only two genes, namely *DREB3* and *BTB/POZ* and *MATH domain-containing protein 2-like* (*BTB/POZ*), in it and is not connected with other clusters. It is known that expression of *DREB3* is significantly induced in response to salinity, drought, low temperature, and H_2_O_2_ in tomato [65]. However, the connection of DREB3 with BTB/POZ is novel. BTB/POZ is known as a transcriptional regulator and is involved in various biological processes. BTB/POZ protein performs different roles, namely ethylene biosynthesis, blue light signal transduction, leaf development, and ABA and SA signaling. A study showed that the MATH domain in BTB/POZ is used to assemble with the members of the ethylene response factor/Apetala2 TF [66]. Plants possess a large number of BTB/POZ proteins, and the functions of only a few are known. Therefore, it is possible that BTB/POZ proteins also interact with *DREB3* to promote defense response against abiotic stresses.

Together, our results suggest that βCC reprograms the transcriptome of the tomato plants to prepare them for various environmental stresses. Additionally, KEGG enrichment analysis and co-functional study of upregulated genes that code for different transcription factors such as *MYC*, *ERF*s, and *DREB3* explained that βCC precisely regulates those genes that are pioneers in acclimation to abiotic stresses, such as heat, drought, and oxidation, and biotic stresses, such as fungal and herbivore attack; however, it is not clear if it can elicit major phytohormone signaling.

## 4. Materials and Methods

### 4.1. Plant Material, Growth Conditions, and βCC Treatment

Seeds of tomato var. Pusa Ruby were collected from the local nursery and germinated on cocopeat. Fifteen-day-old seedlings were transferred to 9 cm × 9 cm pots containing soil: cocopeat: vermiculite (5:4:1) and grown in the polyhouse of the Department of Botany, Savitribai Phule Pune University (Pune, India) at 25–27 °C, 55% relative humidity, and under the natural light regime. Previously, we found that exogenous application of 1 mL pure βCC can increase drought tolerance traits and resistance against *Spodoptera littoralis* larvae in tomato and Arabidopsis plants, respectively [3,11]. Therefore, five-week-old tomato plants were kept in a transparent, closed glass container, where a watch glass containing 1 mL pure βCC (Sigma-Aldrich, Steinheim, Germany) was introduced inside this closed container and allowed to volatilize for 4 h, as described previously [3,10,11]. Plants treated with distilled water were used as control. After 4 h, all the leaves (5–6) were harvested in liquid nitrogen and stored at −80 °C until further use.

### 4.2. RNA Extraction, cDNA Library Preparation, and RNA Sequencing

Total RNA was extracted from control and βCC-treated leaves of tomato plants using TRI reagent (Sigma-Aldrich, Steinheim, Germany) according to the manufacturer’s protocol. After checking the integrity and quantity of RNA samples, mRNA was enriched and purified for the preparation of the cDNA library. Reverse transcription reaction was followed by the end repair and A-tailing of cDNA strands to which adapters were ligated and PCR amplification was carried out. Finally, the strands were separated and cyclized for DNA nanoball synthesis. The sequencing of the DNA nanoballs was done in a BGISEQ-500 platform. RNA sequencing data were obtained from three biological replicates from each treatment (control and βCC-treated). The raw data were submitted to the National Center for Biotechnology Information (NCBI) Sequence Read Archive (SRA) under the bioproject accession number PRJNA771784.

### 4.3. Quality Check and Analysis of RNA Sequence Data

Raw read data of the RNA sequencing were obtained in the FastQ file, which is analyzed for quality control of sequencing data using the FastQC tool. The error in per base sequence content was corrected by the Trimmomatic flexible read trimming tool. The resulting trim reads were aligned with the reference genome of the tomato (Solanum_lycopersicum.SL3.0.dna.toplevel.fa), which is available in Ensembl database (http://plants.ensembl.org/Solanum_lycopersicum/Info/Index, accessed on 9 November 2021). The clean reads were mapped using RNASTAR (Spliced Transcripts Alignment to a Reference) Aligner. This tool achieves highly efficient mapping by performing a two-step process—(1) seed searching and (2) clustering, stitching, and scoring—and then generates a BAM file. The BAM files were further analyzed for the read count of alignment files using the Feature Count tool and reference transcriptome annotation file (.gtf format) downloaded from Ensembl database. This analysis generated count tables which were used for the differential gene expression analysis using the DESeq2 tool. Differentially expressed genes were detected by mean normalized counts averaged over all samples from both conditions, and the logarithm (to the base 2) of the fold change (log_2_ FC) was determined. Statistical significance of the FC was determined by Wald’s statistics with the Benjamini–Hochberg corrections to control false discovery rate (FDR). In a particular comparison of gene expression between control and treated samples, if the log_2_ FC of the gene was >1 and their false discovery rate (FDR) was <0.05, the gene was considered as significantly differentially expressed.

### 4.4. Quantitative Real-Time PCR (RT-PCR)

Total RNA was extracted from 100 mg pulverized tissue using TRI reagent (Sigma-Aldrich, Steinheim, Germany) according to the manufacturer’s protocol. An amount of 500 ng of total RNA was used to synthesize cDNA using iScript^TM^ cDNA synthesis kit (BioRad Laboratories, Inc., Foster City, CA, USA). Quantitative RT-PCR was conducted with four biological replicates from each treatment (control and βCC-treated) on the CFX96 RT-PCR detection system (BioRad Laboratories, Inc., Singapore). A real-time PCR kit (Takara Bio Inc., Shiga, Japan) was used for the amplification using a program operated at 95 °C for 3 min, 40 cycles of 95 °C for 10 s, and 52–60 °C for 30 s. Primers used for the amplification showed a primer efficiency of 88–105%. Different endogenous controls, namely *glyceraldehyde phosphate dehydrogenase* (*gapdh*), *Elongation factor 1* (*ef1*), and *β-tubulin*, were amplified on the cDNA of control and treated samples. Among them, *β-tubulin* and *gapdh* showed the most consistent expression; therefore, *gapdh* was used as an endogenous control to determine relative expression of the candidate genes. Sequences of all primers are provided in Supplementary Data S7. The data were analyzed using a standard curve method as described in Larinov et al. [67].

### 4.5. Analysis of GO and Its Enrichment, Functional Annotations of Differentially Expressed Genes (DEGs), and Enrichment of KEGG

Ontology of DEGs concerning GOBP, GOCC, and GOMF of the translated proteins was retrieved using Protein Knowledgebase database (UniProtKB; https://www.uniprot.org/, accessed on 9 November 2021) and genomic database resources such as EnsemblPlants (https://plants.ensembl.org, accessed on 9 November 2021) and Gramene (https://www.gramene.org/, accessed on 9 November 2021). The annotations of up- and downregulated genes were analyzed separately for their enrichment using the tool ShinyGO v0.66 [68]. Significantly enriched GO terms (FDR < 0.05) were determined and classified into functional categories whose interrelations are visualized as an enrichment network generated at edge cut-off 0.2. Further, functions of upregulated DEGs were predicted with the help of databases and scientific literature specific to tomato. Genes that are not characterized for tomato were predicted for their functions by referring to orthologous genes annotated in Arabidopsis. Functional predictions were further supported by the determination of a molecular class of proteins using the genome-centric portal Ensembl-Plants for tomato genome assembly SL3.0 and co-expressed pathway database (CoxPathDB) for Tomato v.2.1.0 (http://cox-path-db.kazusa.or.jp/tomato/, accessed on 9 November 2021). Similar to the GO enrichment analysis, KEGG enrichment analysis was performed using the same software and parameters.

### 4.6. Generation of DEG Network

To see the comprehensive picture of the molecular orchestra organized by exogenous βCC treatment, a network of upregulated DEGs was generated using TomatoNet, a network-based gene prioritization server for tomato [18]. The network is visualized in Cytoscape_v3.8.2, a network visualization software [69]. A receiver operating characteristic (ROC) curve was generated to score the effectiveness of a network generated at FDR < 0.05.

### 4.7. Statistical Analysis

The statistical significance of the fold change was determined by Wald’s statistics with the Benjamini–Hochberg corrections. The significance of the GO and KEGG enrichment was determined by hypergeometric test using ShinyGO v0.66 [68]. Student’s *t*-test was used to analyze the q-RT PCR data using the PAST (version 3.22) freeware for Microsoft Windows [70].

## Data Availability

The sequencing reads generated for the current study were deposited in the NCBI Sequence Read Archive (SRA) under the bioproject accession number PRJNA771784.

## Supplementary Materials

The following are available online at https://www.mdpi.com/article/10.3390/plants10112465/s1, Figure S1: GO enrichment of βCC-induced downregulated genes and interrelationship of the functional groups, Data S1: βCC-induced coding DEGs and their description, Data S2: GO annotations of coding genes, Data S3: GO enrichment of upregulated genes, Data S4: Functional prediction of upregulated genes, Data S5: KEGG enrichment of upregulated stress-responsive genes, Data S6: Co-functional analysis of upregulated stress-responsive genes, Data S7: Sequences of primers used in quantitative real-time (q-RT) PCR.
