# Supplementary material for: β-Cyclocitral, a Master Regulator of Multiple Stress-Responsive Genes in Solanum lycopersicum L. Plants
Source: Plants (Basel). 2021 Nov 15;10(11):2465. doi: 10.3390/plants10112465 (PMC8618229; doi:10.3390/plants10112465)
Supplement: Supplementary file 1 [file plants-10-02465-s001.zip › Supplemental Data S6.pdf]

Supplemental Data S6: Summary of genes present in co-functional network of stress-responsive upregulated genes. Among 92 stress-responsive genes, 18 genes are showing co-expression. The table summarizes the query genes that are part of the co-functional network with their description, number of query genes connected to each one of them along with respective gene IDs, and score of their connection with query genes.

Rank	Query genes' ID	Description	Linked query genes	Linked query gene ID	Score
1	Solyc09g075950.1	Heat shock 70 kDa protein 8	6	Solyc03g120090.1<br>Solyc04g078420.1<br>Solyc09g008970.1<br>Solyc09g075470.2<br>Solyc10g084170.1<br>Solyc12g009240.1	17.47
2	Solyc06g074030.1	Probable CCR4-associated factor 1 homolog 9	6	Solyc02g091180.1<br>Solyc03g093610.1<br>Solyc06g049020.1<br>Solyc08g082090.1<br>Solyc10g006130.1<br>Solyc10g050970.1	14.21
3	Solyc08g082090.1	Uncharacterized protein LOC101261732	6	Solyc02g088090.1<br>Solyc02g091180.1<br>Solyc03g093610.1<br>Solyc06g074030.1<br>Solyc08g076930.1<br>Solyc10g006130.1	13.22
4	Solyc10g006130.1	Ethylene-responsive transcription factor 3	4	Solyc02g091180.1<br>Solyc06g049020.1<br>Solyc06g074030.1<br>Solyc08g082090.1	8.95
5	Solyc02g091180.1	Uncharacterized protein LOC104645686	4	Solyc06g074030.1<br>Solyc08g076930.1<br>Solyc08g082090.1<br>Solyc10g006130.1	8.87
6	Solyc03g120090.1	Pyridoxal 5'-phosphate synthase-like subunit PDX1.2	2	Solyc09g075950.1<br>Solyc10g084170.1	5.39
7	Solyc12g009240.1	Ethylene-responsive transcription factor ERF017	2	Solyc09g075950.1<br>Solyc10g050970.1	4.73
8	Solyc10g084170.1	BAG family molecular chaperone regulator 5, mitochondrial-like	2	Solyc03g120090.1<br>Solyc09g075950.1	4.56

9	Solyc09g075470.2	dnaJ homolog subfamily B member 6-like	1	Solyc09g075950.1	4.35
10	Solyc04g072900.1	Dehydration-responsive element binding protein 3	1	Solyc09g072980.2	4.28
11	Solyc09g072980.2	BTB/POZ and MATH domain-containing protein 2-like	1	Solyc04g072900.1	4.28
12	Solyc03g093610.1	Ethylene-responsive transcription factor 1	2	Solyc06g074030.1<br>Solyc08g082090.1	3.36
13	Solyc08g076930.1	Transcription factor MYC2	2	Solyc02g091180.1<br>Solyc08g082090.1	3.31
14	Solyc06g049020.1	Uncharacterized protein LOC101254906	2	Solyc06g074030.1<br>Solyc10g006130.1	3.17
15	Solyc04g078420.1	Transcription factor MYB44	1	Solyc09g075950.1	2.8
16	Solyc10g050970.1	Ethylene-responsive transcription factor ERF109-like	2	Solyc06g074030.1<br>Solyc12g009240.1	2.17
17	Solyc09g008970.1	Uncharacterized protein LOC101245195	1	Solyc09g075950.1	2.13
18	Solyc02g088090.1	Probable calcium-binding protein CML30	1	Solyc08g082090.1	1.15
