# Supplementary material for: β-Cyclocitral, a Master Regulator of Multiple Stress-Responsive Genes in Solanum lycopersicum L. Plants
Source: Plants (Basel). 2021 Nov 15;10(11):2465. doi: 10.3390/plants10112465 (PMC8618229; doi:10.3390/plants10112465)
Supplement: Supplementary file 1 [file plants-10-02465-s001.zip › Supplemental Data S7.pdf]

Table S1. Sequences of primers used in Real time-quantitative PCR (RT-qPCR). Primers are designed on the cDNA of *Solanum lycopersicum* and used for RT-qPCR amplification for the validation of differentially expressed genes.

Gene ID	Primer Sequence	Description	Tm (°C)	Product size (bp)
Solyc08g076930.1	5′TACTTCCAGGGGAAGCAATG3′<br>3′GACGTGATTCAATGGCTCCT5′	MYC2	60	210
Solyc04g072900.1	5′TGGAGGAAGAGGAGAGCAAA3′<br>3′GAGAGGCCCAATCAATTTCA5′	Dehydration-responsive element binding protein 3 (DREB)	60	238
Solyc09g015770.3	5′TGATGGTCATGCTTGGAGAA3′<br>3′GCTTTGCAAGTGTGATGTCC5′	WRKY 3	60	199
Solyc10g086410.3	5′TGGCCTAAATGTGATGCGTA3′<br>3′CCAGCAGTAGCCTTCACCTC5′	Heat shock cognate70 (HSC70)	55	183
Solyc09g075440.3	5′AGGAAGAACATTGGCATTGG3′<br>3′GCATGTATTTCCCAGCAGGT5′	Ethylene Receptor	60	238
Solyc02g094040.3	5′CATTCATCGCCGGTTGACAT3′<br>3′TGCTTGTTCTTGGCCTTCAC5′	Lipase	54	204
Solyc05g051660.1	5′AACACCGCACCCAATCAAAA3′<br>3′GTGCATCCCAACAATCCTGG5′	Acylsugar acylhydrolase 1 (ASH1)	54	202
Solyc02g080530.3	5′CAAGCGCATCAGACACATCT3′<br>3′GCCACCAGTGGAGTTGAAAT5′	Peroxidase	60	165
Solyc11g072630.2	5′CCGGAGCTTTTGTTGAACTC3′<br>3′GTGGGAGTTGCCTGACGTAT5′	Mitogen-activated protein kinase 3 (MPK3)	52	185
Solyc08g006410.3	5′CAAAGGGAAAAGGGATGGTT3′<br>3′ACCCCTATGCCAAGTTCCTC 5′	Glycosyltransferase	60	194
House keeping gene	5′GCCATCAAGGAGGAATCTGA3′<br>3′CATTCGTTGTCGTACCATGC5′	Glyceraldehyde 3-phosphate dehydrogenase (GAPDH)	60	173
