# Supplementary material for: β-Cyclocitral, a Master Regulator of Multiple Stress-Responsive Genes in Solanum lycopersicum L. Plants
Source: Plants (Basel). 2021 Nov 15;10(11):2465. doi: 10.3390/plants10112465 (PMC8618229; doi:10.3390/plants10112465)
Supplement: Supplementary file 1 [file plants-10-02465-s001.zip › Supplemental Figure S1.pdf]

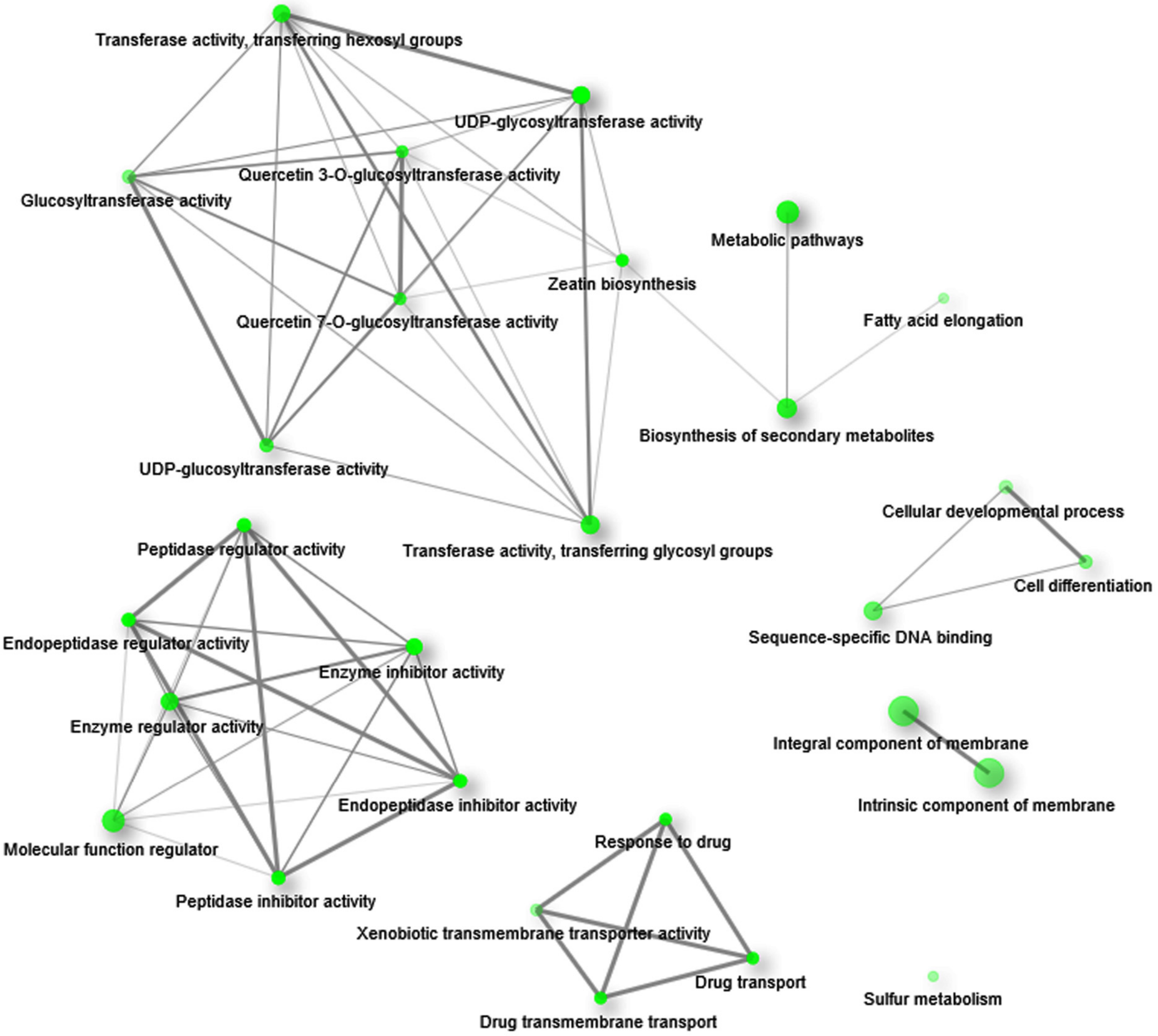

Figure S1. GO enrichment of βCC-induced downregulated genes and interrelationship of the functional groups. GO enrichment of downregulated genes showed functional interrelationship among different functional groups. The network represents the top 30 significant functional groups at enrichment FDR< 0.05. The network showed scattered clusters of different functional groups. Green nodes represent the functional groups. The size of the node is proportional to the number of genes and the intensity of the green color is proportional to the enrichment value at FDR< 0.05. Grey lines (edges) that are joining the nodes are showing functional interrelations between the nodes. For better visualization the position of the nodes were rearranged.
